# Systematic Identification of Novel, Essential Host Genes Affecting Bromovirus RNA Replication

**DOI:** 10.1371/journal.pone.0023988

**Published:** 2011-08-22

**Authors:** Brandi L. Gancarz, Linhui Hao, Qiuling He, Michael A. Newton, Paul Ahlquist

**Affiliations:** 1 Institute for Molecular Virology, University of Wisconsin–Madison, Madison, Wisconsin, United States of America; 2 Howard Hughes Medical Institute, University of Wisconsin-Madison, Madison, Wisconsin, United States of America; 3 Department of Statistics, University of Wisconsin–Madison, Madison, Wisconsin, United States of America; 4 Department of Biostatistics and Medical Informatics, University of Wisconsin–Madison, Madison, Wisconsin, United States of America; Kantonal Hospital St. Gallen, Switzerland

## Abstract

Positive-strand RNA virus replication involves viral proteins and cellular proteins at nearly every replication step. Brome mosaic virus (BMV) is a well-established model for dissecting virus-host interactions and is one of very few viruses whose RNA replication, gene expression and encapsidation have been reproduced in the yeast *Saccharomyces cerevisiae*. Previously, our laboratory identified ∼100 non-essential host genes whose loss inhibited or enhanced BMV replication at least 3-fold. However, our isolation of additional BMV-modulating host genes by classical genetics and other results underscore that genes essential for cell growth also contribute to BMV RNA replication at a frequency that may be greater than that of non-essential genes. To systematically identify novel, essential host genes affecting BMV RNA replication, we tested a collection of ∼900 yeast strains, each with a single essential gene promoter replaced by a doxycycline-repressible promoter, allowing repression of gene expression by adding doxycycline to the growth medium. Using this strain array of ∼81% of essential yeast genes, we identified 24 essential host genes whose depleted expression reproducibly inhibited or enhanced BMV RNA replication. Relevant host genes are involved in ribosome biosynthesis, cell cycle regulation and protein homeostasis, among other cellular processes. BMV 2a^Pol^ levels were significantly increased in strains depleted for a heat shock protein (*HSF1)* or proteasome components (*PRE1* and *RPT6*), suggesting these genes may affect BMV RNA replication by directly or indirectly modulating 2a^Pol^ localization, post-translational modification or interacting partners. Investigating the diverse functions of these newly identified essential host genes should advance our understanding of BMV-host interactions and normal cellular pathways, and suggest new modes of virus control.

## Introduction

Viruses survive with limited genetic material by interacting with and exploiting host factors at essentially every replication step [1], [2], [3], [4], [5], [6], [7], [8], [9], [10], [11], [12], [13], [14]. Identifying the host factors and pathways exploited in virus replication and the nature of their contributions and interactions with virus-encoded replication factors represent major challenges and opportunities for understanding and controlling viruses.

Positive-strand RNA viruses comprise over one third of all virus genera and include important human pathogens such as hepatitis C virus, dengue virus, chikungunya virus, and West Nile virus [15]. Brome mosaic virus (BMV), a member of the alphavirus-like superfamily of human, plant, and animal viruses, has been used as a model system to study gene expression, RNA replication and virus-host interactions of positive-strand RNA viruses [1], [2], [8], [10], [16]. BMV has three genomic RNAs and one subgenomic (sg) mRNA. Genomic RNAs 1 and 2 encode the multifunctional replication proteins 1a and 2a polymerase (2a^Pol^), respectively, which are required for RNA replication [17], [18], [19]. Genomic RNA3 encodes the 3a movement protein, required for infection spread in plants. A sg mRNA, RNA4, encodes the viral coat protein and is produced from the (−)RNA3 replication intermediate. BMV RNA replication and encapsidation can be recapitulated in the yeast *Saccharomyces cerevisiae* by expressing the viral replication and/or capsid proteins together with at least one genomic RNA template [20], [21], [22]. The ability of BMV to duplicate nearly all major replication features of its natural plant hosts in yeast, combined with yeast genetics, has advanced our understanding of BMV replication and virus-host interactions [1], [2], [9].

Previously, we tested deletions of nearly all non-essential yeast genes (∼80% of the yeast genome) and identified 99 genes, that when deleted, altered BMV replication, revealing the involvement of many novel host pathways in viral replication, transcription, and translation [9]. However, classical yeast genetics and other approaches have demonstrated that genes essential for cell growth also make major contributions to BMV RNA replication [23], [24], [25], [26]. To more globally identify additional essential host factors critical for BMV RNA replication, we assayed a doxycycline (dox)-repressible library of ∼900 yeast strains, each of which allows repressing the expression of a selected essential gene by adding dox to growth media [27]. Using this genome-wide approach, we identified 24 essential host factors whose repressed expression reproducibly altered BMV RNA replication. These host factors are involved in protein homeostasis, protein trafficking, and translation, among others. The results presented here, in conjunction with previously identified host factors [2], [8], [10], [24], [25], provide a more complete understanding of cellular pathways utilized by BMV. Dissecting the role of these essential host genes in virus replication should significantly advance our understanding of basic virus biology and virus-host interactions. Additionally, these results may lay a foundation for extending such studies to other virus groups, thus potentially identifying common cellular pathways that could be targeted for the development of broad-spectrum antivirals.

## Methods

### *S. cerevisiae* Strains and Plasmids

The yeast Tet-Promoter Hughes Collection of essential yeast strains was purchased from Open Biosystems (Huntsville, AL). The tet-promoter mutant strains (designated here with the prefix P*_TET_*) were provided in the haploid R1158 background (*MATa URA3::CMV-tTA MATa his3-1 leu2-0 met15-0*), which was constructed by a one-step integration of the cytomegalovirus (CMV) promoter-driven tTA* transactivator at the *URA3* locus [28]. The kanR-tetO7-TATA was then integrated into the promoter of a different essential gene in strain R1158, allowing the repression of essential gene expression upon the addition of dox to growth medium [27].

pB12VG1 expresses BMV 1a and 2a^Pol^ from the *GAL1* and *GAL10* promoters, respectively [9]. pB3BG29, based on pB3Rluc [9], uses a truncated *GAL1* promoter (GALL [29]) to express RNA3 with the coat protein ORF replaced by the *Renilla* luciferase (Rluc) ORF (from pRL-null; Promega, Madison, WI). pB3BG29 also expresses the firefly luciferase (Fluc) ORF (from pGL3-Basic; Promega, Madison, WI) from the *GAL10* promoter. To construct pB3BG29, the *Age*I-*Aat*II RNA3/Rluc-containing fragment of pB3Rluc [9] replaced the *Age*I-*Aat*II FHV RNA1/Rluc-containing fragment of pBDL250-Ren [B. Lindenbach, unpublished]. pB3MS82 expresses a BMV RNA3 derivative in which the coat protein gene has a four-nucleotide insertion and a point mutation, abolishing expression of the coat protein [30]. The use of pB3MS82 in this study, as in many other studies [8], [19], [30], [31], [32], [33], allows analysis of RNA3 and RNA4 levels while avoiding any possible effects of coat protein expression and RNA encapsidation. pB12VG1 and pB3BG29 were used in reporter gene-based primary screens and pB12VG1 and pB3MS82 were used in secondary validation testing by Northern blotting.

### Yeast transformation and growth

96-well yeast transformations were based on a one-step procedure [34]. The P*_TET_* essential yeast strains were grown to saturation overnight at 30°C in 96-well plates (1 ml per well). The cells were pelleted, suspended in 100 µl of transformation mix (0.18 M LiAc, pH 5.5, 36% polyethylene glycol-3350, 90 mM DTT, 0.5 mg/ml sheared salmon sperm DNA, and 20 µg/ml of each plasmid) per well and incubated at 30°C for 60 min. Cells then were heat shocked at 42°C for 20 min, pelleted, re-suspended in 20 µl sterile water per well and 10 µl was plated on solid media. Transformants were selected by complementation of auxotrophic markers. 96-well plates of transformed yeast were re-formatted to contain 48 strains in duplicate per plate so that strains could be analyzed in the absence of dox (allowing essential gene expression) or in the presence of dox (repressing essential gene expression). Strains containing BMV expression plasmids were grown overnight in medium containing raffinose, subcultured to a starting OD_600_ = 0.1 in medium containing raffinose ±10 µg/ml dox, grown for 24 hr subcultured to a starting OD_600_ = 0.1 in medium containing galactose ±10 µg/ml dox. Cells were analyzed at 24 hr and 48 hr post gal-induction of virus expression. Strains were grown in 96-well plates for luciferase assays and 14 ml culture tubes for Northern analysis.

### RNA Analysis

For 96-well Fluc assays, 2.5 µl of cells were lysed in 1× Passive Lysis Buffer (Promega, Madison, WI), 25 µl of Luciferase Assay Substrate (Promega, Madison, WI) was injected and read for 1 s with a 1.6 s delay using a VictorV (PerkinElmer, Waltham, MA). For 96-well Rluc assays, 5 µl of cells were lysed in 1× Passive Lysis Buffer, 25 µl of *Renilla* Luciferase Assay Substrate (Promega, Madison, WI) was injected and read for 1 s with a 1.6 s delay using a VictorV. To allow comparison between plates, the median of untreated samples and the median of dox-treated samples were calculated for each plate. Each untreated sample was then normalized to the untreated median whereas each dox-treated sample was normalized to the dox-treated median. For each pass of the 741 P*_TET_* strains, we calculated BMV-directed Rluc expression as [Rluc_dox-treated_/Fluc_dox-treated_] normalized to the dox-treated median and [Rluc_untreated_/Fluc_untreated_] normalized to the untreated median. The dox-treated to untreated ratio of ratios was calculated and converted to fold change. High-throughput isolation of total RNA from yeast cells was performed as previously described [35]. Northern blotting was performed as previously described [36] except that 2 µg RNA was separated in 1% (wt/vol) agarose-MOPS (morpholinepropanesulfonic acid)-formaldehyde gels. RNAs were detected using ^32^P-labeled probes specific for positive- or negative-strand BMV RNA3 and RNA4 as previously described [24]. The 18S rRNA probe was derived from pTRI RNA 18S templates (Ambion, Austin, TX). Probes were synthesized using an Epicenter Riboscribe probe synthesis kit (Madison, WI) with the appropriate enzyme, i.e., T7 or or SP6 polymerase. Northern blots were imaged on a Typhoon 9200 instrument (Amersham Biosciences, Piscataway, NJ) and band intensities were analyzed with ImageQuant software (Molecular Dynamics, Piscataway, NJ).

### Protein extraction, Western blotting, and total protein analysis

Total protein was extracted as previously described [24] and equal volumes of cell lysates were separated on 4–15% Criterion™ TGX™ precast polyacrylamide gels (Bio-Rad, Hercules, CA). Proteins were transferred to PVDF membrane and expression of target proteins was detected with the following antibodies and dilutions: rabbit anti-BMV 1a at 1∶10,000, mouse anti-BMV 2a^Pol^ at 1∶3,000, and mouse anti-Pgk1p (Molecular Probes, Carlsbad, CA) at 1∶10,000 using HRP-conjugated secondary antibodies (Thermo Scientific, Rockford, IL) and Supersignal West Femto substrate (Thermo Scientific, Rockford, IL).

### Statistical analysis

The tools in R statistical package (version R-2.11.1) (http://www.r-project.org/) [37] was used for statistical analysis of BMV RNA replication data obtained by Northern blotting. Log transformation was applied to [RNA4/18S rRNA]_dox-treated_/[RNA4/18S rRNA]_untreated_ ratios from Northern blot data, where 18S rRNA served as normalization standard. One-sided *t*-statistics were used to identify the dox-treated strains whose RNA replication was statistically significantly altered compared to untreated strains. *p*-values from *t*-statistics were converted to *q*-values to control for false discovery rate [37].

## Results

### Identification of Essential Host Genes Affecting BMV RNA Replication

To systematically identify novel, essential host genes affecting BMV RNA replication, we screened a dox-repressible library of essential yeast strains [27]. This collection contains ∼900 yeast strains, each with a single endogenous essential gene promoter replaced by a tetracycline-repressible promoter [27]. Upon the addition of the tetracycline derivative dox to growth media, expression of the essential gene is repressed, and the protein depleted. This library was used to identify changes in BMV RNA replication after repression (dox-treated) relative to continuous expression (untreated) of a specific essential host gene.

**Figure 1:**
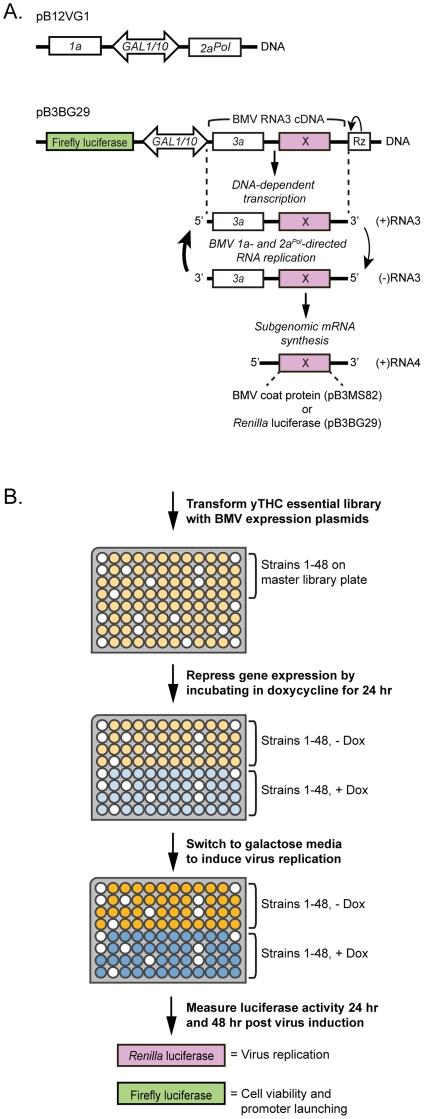
Yeast genetic screen used to identify essential host factors affecting BMV RNA replication. (A) BMV expression plasmids. pB12VG1 expresses replication factors 1a and 2a^Pol^. pB3BG29 expresses Fluc and RNA3. BMV-specific RNA-dependent RNA replication and subgenomic mRNA synthesis is initiated from a cDNA derivative of RNA3. DNA-dependent transcription produces an initial (+)RNA3 transcript that serves as a template for 1a- and 2a^Pol^-dependent RNA3 replication and sgRNA4 synthesis via a (−)RNA3 intermediate. X, the BMV coat protein gene or any gene replacing it, such as Rluc (used here); *GAL1*/*GAL10*, yeast promoters, Rz, self-cleaving ribozyme. (B) 892 yeast strains, each with a single essential gene promoter replaced by a doxycycline (dox)-repressible promoter, were transformed with BMV expression plasmids. White wells indicate strains that did not transform. Transformants were re-formatted on 96-well plates with duplicates of each strain present on the same plate, allowing untreated and dox-treated strains to be directly compared. Strains were grown in raffinose-containing selective medium lacking dox (allowing essential gene expression) or containing 10 µg/ml dox (repressing essential gene expression) for 24 hr to allow for initial depletion of the essential gene mRNA and protein turnover in dox-treated strains. After this 24 hr treatment, strains were sub-cultured into galactose-containing selective medium ±10 µg/ml dox to induce expression of BMV components and subsequent viral RNA replication. Viral RNA replication was quantitated with a chemiluminescent *Renilla* luciferase assay at 24 hr and 48 hr post-virus induction. Cell viability and promoter launching were monitored with a chemiluminescent firefly luciferase assay at 24 hr and 48 hr post-virus induction. Two independent analyses of the library were performed.

To assess the role of essential genes in BMV RNA replication, we co-transformed each of the ∼900 strains with BMV expression plasmids pB12VG1 and pB3BG29 (Fig. 1A). pB12VG1 expresses BMV RNA replication proteins 1a and 2a^Pol^ [9]. pB3BG29 expresses an Rluc reporter-expressing BMV RNA3 cDNA derivative and, as an expression control, the Fluc reporter gene (Fig. 1A). DNA-dependent transcription produces an initial (+)RNA3 transcript that serves as a template for 1a- and 2a^Pol^-dependent RNA3 replication and sgRNA4 synthesis via a (−)RNA3 intermediate (Fig. 1A). Because sgRNA4, which encodes the coat protein, is derived from the (−)RNA3 intermediate, its synthesis depends on, and can serve as a reporter for, BMV RNA replication [20]. Accordingly, in pB3BG29 we replaced the coat protein ORF with the Rluc reporter gene, so that luciferase assays could be used as a rapid measure of RNA4 production and expression (Fig. 1A). A similar approach was used for primary screening of the yeast non-essential gene deletion library [9]. Of the 892 dox-repressible strains transformed, 151 did not produce colonies after repeated transformation attempts (Table S1), suggesting that the transformation process or expression of the viral constructs significantly impacted the fitness of these strains.

The remaining 741 transformants were re-formatted on 96-well plates such that duplicates of each strain were present on the same plate (Fig. 1B), allowing untreated and dox-treated strains to be directly compared for changes in RNA replication. It is important to note for this library that expression levels from the substituted TET promoter are often different than expression levels from the endogenous gene promoter [27]. Therefore, to test and to control for possible changes in viral RNA replication and/or gene expression, each treated strain was compared to its untreated counterpart rather than to the parental wild type strain. Strains were grown in raffinose-containing selective medium lacking dox (allowing essential gene expression) or raffinose-containing selective medium containing 10 µg/ml dox (repressing essential gene expression) for 24 hr to allow for initial depletion of the essential gene mRNA and protein turnover in strains treated with dox. After this 24 hr treatment period, strains were sub-cultured into galactose-containing selective medium ±10 µg/ml dox to induce the expression of BMV components and subsequent viral RNA replication in the continued expression (untreated) or repression (dox-treated) of essential host factors. At 24 hr and 48 hr post-virus induction, Rluc expression was measured as a readout of BMV RNA3 replication and sgRNA4 synthesis. Because of expected differences in the kinetics of gene product depletion and their specific and non-specific effects on cell growth and BMV RNA replication, we measured Rluc at 24 hr and 48 hr post-virus induction for every strain. To monitor any potential adverse effects of dox on the viability of these yeast strains and to ensure that *GAL* promoter induction was effective, *GAL* driven Fluc expression, which is independent of BMV RNA replication (Fig. 1A), was also assayed at 24 hr and 48 hr post-virus induction (Fig. 1B). Two independent analyses of the entire library were performed.

Since ∼70% of the strains in this library exhibit growth defects in the presence of dox (∼20% of which exhibit growth defects even in the absence of dox, presumably as a result of endogenous promoter replacement) [27] and because BMV RNA replication levels are often non-specifically enhanced in slow-growing cells, stringent growth requirements were employed to minimize false positives. Additionally, because we expected some strains to exhibit either immediate dox-induced growth defects and/or transcriptional defects from the *GAL* promoter, Fluc values in untreated vs. dox-treated strains were closely monitored. Accordingly, to be included in the final data analysis for potentially specific effects on BMV replication, each strain was required to: 1) double at least twice between 0–24 hr in galactose-containing media; 2) double at least one additional time between 24–48 hr in galactose-containing media; 3) and have an Fluc_dox-treated_ value within 20% of its Fluc_untreated_ value. As a reference, wild type strain R1158 doubled 3 times between 0–24 hr and 2 times between 24–48 hr and had comparable Fluc_untreated_ and Fluc_dox-treated_ values. Strains that did not meet these growth or Fluc value requirements in both passes were excluded from further analysis (Tables S2 and S3). The remaining strains that satisfied these growth and Fluc requirements and were included in analysis (Table S4) showed >90% overlap between pass 1 and 2 at the 24 hr time point and >80% overlap between passes at the 48 hr time point, confirming good reproducibility of screen conditions (e.g. growth, dox-treatment, etc.) and assay performance.

From the analyzed strains (Table S4), we identified 42 essential yeast genes that, when dox-depleted, altered BMV-directed Rluc expression at least 6-fold in both passes at the same time point (Tables 1 and 2). A more stringent 6-fold cutoff was used in the primary screen in response to our observation that some strains in this essential gene library showed more luciferase assay variability than in our previous screen of non-essential genes. This increased variability is likely due to the fact that these genes are essential for cell growth. Thus, upon dox-induced repression of these genes, the experiment is a race between specific effects of the relevant gene on virus replication and the nonspecific, general suppression of cell growth and viability that eventually occur with each strain. Therefore, the results are more subject to variations due to small changes in growth conditions or timing of the experiments. Accordingly, a 6-fold cutoff was employed to limit the inclusion of false positives.

**Table 1:** Genes whose repression was associated with ≥6-fold enhanced BMV-directed Rluc expression in both primary screen passes.

		Fold increase in Rluc expression	
ORF	Gene	Pass 1	Pass 2
YLR359W	*ADE13*	46	57
YBR070C	*ALG14*	11	14
YDL132W	*CDC53*	8.4	6.3
YOR204W	*DED1*	180	140
YKL078W	*DHR2*	8.3	8.0
YLR129W	*DIP2*	440	160
YMR128W	*ECM16*	12	7.4
YLR274W	*MCM5*	33	19
YGR103W	*NOP7*	46	78
YGR119C	*NUP57*	30	19
YOR122C	*PFY1*	7.9	7.5
YLR196W	*PWP1*	11	28
YOL094C	*RFC4*	21	14
YNL207W	*RIO2* a	16	12
YGL044C	*RNA15*	41	12
YOR340C	*RPA43*	17	6.3
YKR008W	*RSC4*	250	310
YPL124W	*SPC29*	13	10
YGR116W	*SPT6*	45	17
YKL018W	*SWD2*	48	9.1
YDR324C	*UTP4*	12	11
YJL069C	*UTP18*	11	50
YGR251W	N/A^b^	33	150

a *RIO2* was identified at the 24 hr time point and all other genes were identified at the 48 hr time point.

b ORF not annotated in *Saccharomyces* Genome Database.

**Table 2:** Genes whose repression was associated with ≥6-fold inhibited BMV-directed Rluc expression in both primary screen passes.

		Fold decrease in Rluc expression	
ORF	Gene	Pass 1	Pass 2
YKL112W	*ABF1*	15	9.1
YER168C	*CCA1*	7.7	7.6
YFR028C	*CDC14* a	12; 9.2	6.0; 7.8
YNR038W	*DBP6*	26	9.9
YPL266W	*DIM1*	36	70
YDR141C	*DOP1*	6.2	46
YJR017C	*ESS1*	22	22
YOL133W	*HRT1*	7.2	6.2
YGL073W	*HSF1* b	37	17
YGL018C	*JAC1*	15	45
YAL033W	*POP5*	17	12
YER012W	*PRE1* a	21; 8.4	13; 19
YML046W	*PRP39*	9.4	49
YGL048C	*RPT6*	15	52
YKL125W	*RRN3*	13	76
YGR245C	*SDA1*	22	14
YDR472W	*TRS31*	7.5	22
YDR327W	N/A^c^	460	160
YOR262W	N/A^b^ *^, ^* c	9.8	13

a *CDC14* and *PRE1* were identified at both the 24 hr and 48 hr time points and data for both time points is listed as “24 hr; 48 hr”.

b *HSF1* and YOR262W were identified at the 24 hr time point and all other genes were identified at the 48 hr time point.

c ORF not annotated in *Saccharomyces* Genome Database.

In principle, altered BMV-directed Rluc expression observed in the 42 candidate genes might result from general defects in RNA4 translation or viral RNA synthesis, or from selective defects in replication, expression or function of the Rluc reporter gene. To identify genes that specifically affected BMV RNA synthesis and/or accumulation, secondary validation testing of the 42 candidate genes was performed using Northern blotting to analyze RNA3 replication and RNA4 production, as described in the next two sections.

### Dox-repression of 19 essential genes facilitated BMV RNA accumulation

Of the 42 candidate genes identified in the reporter gene-based primary screens, 23 were genes whose repression enhanced BMV-directed Rluc expression relative to Fluc expression at least 6-fold in both passes at the same time point (Table 1). For secondary validation tests, these 42 candidate strains were transformed with the 1a- and 2a^Pol^-expressing plasmid pB12VG1 and with a plasmid expressing RNA3 retaining the BMV coat protein ORF (Fig. 1A, pB3MS82). The levels of RNA3 and RNA4 replication products then were measured by Northern blotting. In particular, we used the level of RNA4 relative to 18S rRNA as the primary measure of any dox-induced change in BMV RNA-dependent RNA synthesis, through the ratio [RNA4/18S rRNA]_dox-treated_/[RNA4/18S rRNA]_untreated_. RNA4 was used as the primary measure of BMV RNA synthesis since, unlike RNA3, RNA4 is only produced by viral RNA-dependent RNA synthesis and not also by DNA-dependent transcription (Fig. 1A). Moreover, RNA4 level is the parameter most closely related to the Rluc expression measured in the primary screen (Fig. 1A).

**Figure 2:**
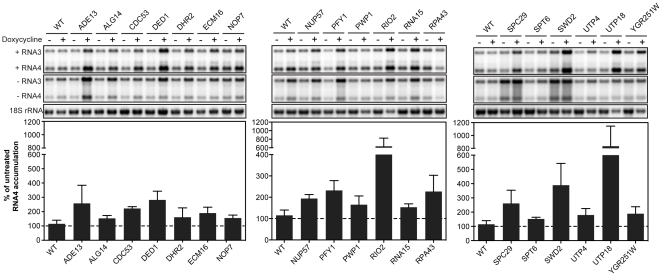
Dox-induced repression of 19 essential yeast genes enhances BMV RNA replication. Total RNA extracts were obtained from wild type R1158 and untreated and dox-treated (10 µg/ml) essential yeast strains expressing BMV 1a, 2a^Pol^ and RNA3. Accumulation of positive- and negative-strand RNA3 and subgenomic RNA4 was detected by Northern blotting using probes specific for BMV RNA3 and RNA4. Equal loading of total RNA was verified by probing for 18S rRNA. Values represent the mean of four independent experiments.

A statistically significant increase in BMV (+)RNA4 accumulation relative to 18S rRNA was confirmed for 19 of the 23 genes (∼83% confirmation) at the commonly applied false discovery rate of 5% (Fig. 2 and Table 3). False discovery rate analysis is a robust statistical method that controls for multiple test variables by calculating an adjusted, more stringent *p*-value, termed a *q*-value [37]. (+)RNA4 levels were enhanced 1.5- to 8-fold in the 19 confirmed strains (Fig. 2 and Table 3). Similarly, levels of (−)RNA3, the replication intermediate that serves as a template for sgRNA4, were increased 1.4- to 5-fold in these strains (Table S5).

**Table 3:** Essential genes whose repression was confirmed to enhance BMV RNA accumulation in secondary validation testing.

ORF	Gene	(+)RNA4 (average % untreated)	q-value
YJL069C	*UTP18*	833±312	0.011
YNL207W	*RIO2*	635±190	0.003
YKL018W	*SWD2*	389±153	0.006
YOR204W	*DED1*	282±61	0.003
YPL124W	*SPC29*	261±93	0.006
YLR359W	*ADE13*	258±126	0.034
YOR122C	*PFY1*	232±46	0.009
YOR340C	*RPA43*	227±76	0.010
YDL132W	*CDC53*	222±13	3.24E-04
YGR119C	*NUP57*	194±19	0.006
YMR128W	*ECM16*	190±41	0.011
YGR251W	N/A^a^	189±45	0.014
YDR324C	*UTP4*	180±49	0.003
YLR196W	*PWP1*	165±41	0.011
YKL078W	*DHR2*	161±65	0.036
YGR103W	*NOP7*	155±21	0.011
YBR070C	*ALG14*	153±20	0.006
YGL044C	*RNA15*	153±16	0.003
YGR116W	*SPT6*	152±12	0.003

a ORF not annotated in *Saccharomyces* Genome Database.

The 19 confirmed dox-repressed genes that enhanced BMV RNA accumulation encode proteins with functions in varied cellular processes, including ribosome biosynthesis (*DHR2*, *ECM16*, *NOP7*, *PWP1*, *RIO2*, *RPA43*, *UTP4*, *UTP18*, and *YGR251w*), cell cycle/DNA maintenance (*ADE13* and *SPC29*), mRNA metabolism (*RNA15*, *SPT6*, and *SWD2*), protein homeostasis (*PFY1*), translation (*DED1*), trafficking (*NUP57*) and lipid synthesis (*ALG14*) (Table 4). Possible relations of these functions to viral replication are considered further in the Discussion.

**Table 4:** Annotated functions of confirmed essential host genes effecting BMV RNA accumulation.

Gene^a^	Gene Description^a^ *^,^* b
Protein Homeostasis	
***ESS1***	**Peptidylprolyl-cis/trans-isomerase; regulates phosphorylation of the RNA polymerase II large subunit**
***HSF1***	**Trimeric heat shock transcription factor**
***JAC1***	**Hsp40/DnaJ family J-protein that functions with Hsp70 in Fe-S cluster biogenesis in mitochondria**
***PRE1***	**Beta 4 subunit of the 20S core of the 26S proteasome**
***RPT6***	**One of six ATPases of the 19S regulatory particle of the 26S proteasome**
*PFY1*	Binds profilin, actin and phosphatidylinositol 4,5-bisphosphate; cytoskeleton organization
Translation	
*DED1*	ATP-dependent DEAD-box RNA helicase, required for translation initiation of all yeast mRNAs
Trafficking	
*NUP57*	Nucleoporin essential for trafficking nucleic acids, proteins, and RNA through the nuclear pore complex
Lipid synthesis	
*ALG14*	Component of UDP-GlcNAc transferase required for dolichyl-linked oligosaccharide synthesis
Ribosome Biosynthesis	
*DHR2*	DEAH-box ATP-dependent RNA helicase, required for 18S rRNA synthesis
*ECM16*	DEAH-box ATP-dependent RNA helicase specific to the U3 snoRNP, required for 18S rRNA synthesis
*NOP7*	Required for large ribosomal subunit maturation; required for exit from G0
*PWP1*	Protein with WD-40 repeats involved in rRNA processing
*RIO2*	Serine kinase involved in the processing of the 20S pre-rRNA into mature 18S rRNA
*RPA43*	RNA polymerase I subunit A43
*UTP4*	Subunit of U3-contaning complexes involved in production of 18S rRNA
*UTP18*	Possible U3 snoRNP protein involved in maturation of pre-18S rRNA
*YGR251w*	Required for 18S rRNA maturation
mRNA Metabolism	
*RNA15*	Component involved in the cleavage and polyadenylation of mRNA 3′ ends
*SPT6*	Transcription elongation factor required for the maintenance of chromatin structure during transcription
*SWD2*	Required for methylation of histone H3 and RNA polymerase II transcription termination
Cell Cycle/DNA Maintenance	
*ADE13*	Adenylosuccinate lyase involved in the purine nucleotide synthesis
*CDC53*	A scaffolding subunit (cullin) for multiple E3 ubiquitin-ligase complexes; regulates G1-S cell cycle progression
*SPC29*	Inner plaque spindle pole body (SPB) component; required for SPB duplication

a Bold font indicates genes whose repression inhibits viral RNA accumulation; non-bold font indicates genes whose repression enhances viral RNA accumulation.

b Based on the *Saccharomyces* Genome Database at http://www.yeastgenome.org/.

### Dox-repression of five essential genes inhibited BMV RNA accumulation

**Figure 3:**
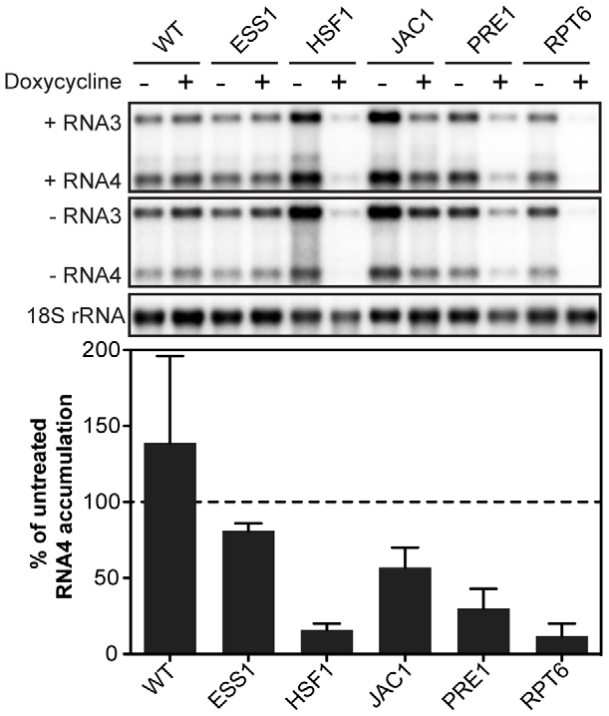
Dox-induced repression of five essential yeast genes inhibits BMV RNA replication. Total RNA extracts were obtained from wild type R1158 and untreated and dox-treated (10 µg/ml) essential yeast strains expressing BMV 1a, 2a^Pol^ and RNA3. Accumulation of positive- and negative-strand RNA3 and subgenomic RNA4 was detected by Northern blotting using probes specific for BMV RNA3 and RNA4. Equal loading of total RNA was verified by probing for 18S rRNA. Values represent the mean of four independent experiments.

The primary screens additionally identified 19 essential host genes that inhibited BMV-directed Rluc expression at least 6-fold in both passes at the same time point (Table 2). Northern blotting confirmed statistically significant decreases in BMV (+)RNA4 accumulation for 5 of 19 genes (∼26% confirmation) at a false discovery rate of 5% (Fig. 3 and Table 5). (+)RNA4 levels were inhibited ∼1.5- to 8-fold in these strains (Fig. 3 and Table 5). In addition to the severe inhibition of (+)RNA4 accumulation in P*_TET_*-*HSF1*, P*_TET_*-*PRE1*, and P*_TET_*-*RPT6*, (−)RNA3 was inhibited 5.5-, 1.8-, and 5.5-fold in these strains, respectively (Fig. 3 and Table S6).

**Table 5:** Essential genes whose repression was confirmed to inhibit BMV RNA accumulation in secondary validation testing.

ORF	Gene	(+)RNA4 (average % untreated)	q-value
YGL048C	*RPT6*	12±8	0.024
YGL073W	*HSF1*	16±4	0.004
YER012W	*PRE1*	30±13	0.024
YGL018C	*JAC1*	57±13	0.029
YJR017C	*ESS1*	81±5	0.024

Interestingly, all five genes that inhibited BMV RNA replication (*ESS1*, *HSF1*, *JAC1*, *PRE1*, and *RPT6*) perform cellular functions that, in various ways, contribute to modulating host protein levels (Table 4).

### BMV 2a^Pol^ protein levels are affected in some dox-repressed strains

**Figure 4:**
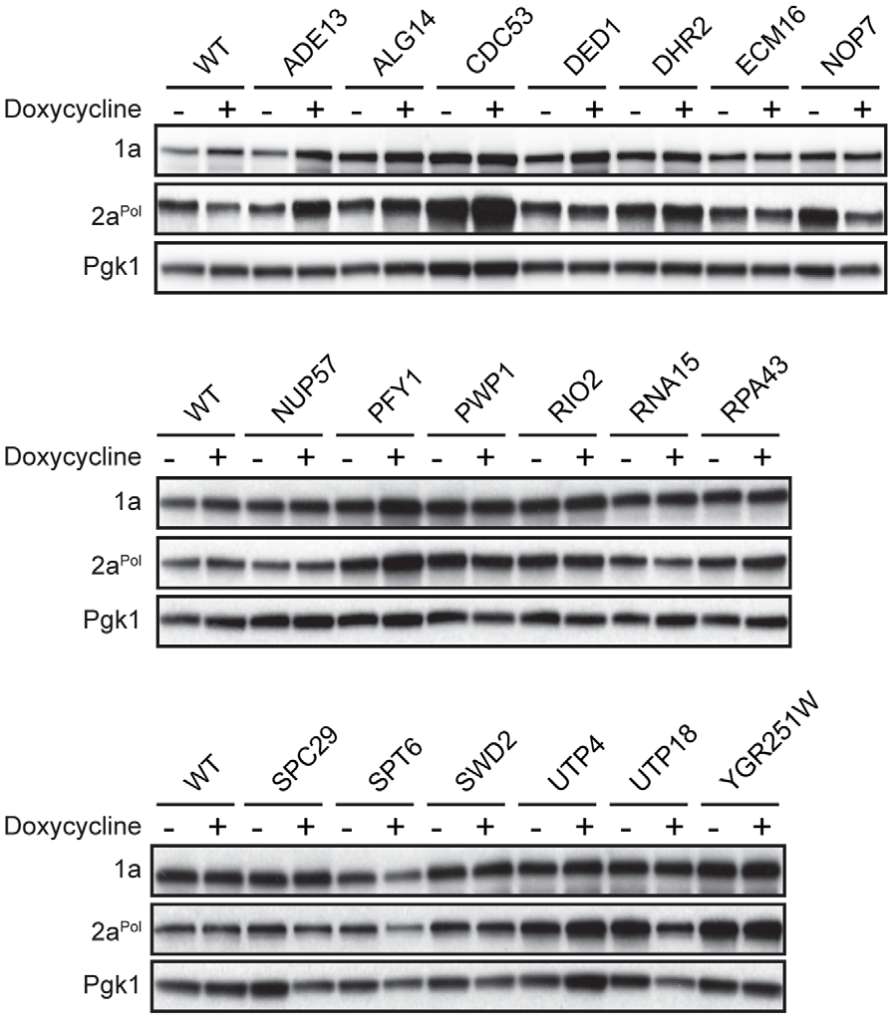
BMV 1a and 2a^Pol^ levels for dox-repressed essential yeast genes with enhanced BMV RNA replication. Accumulation of BMV 1a and 2a^Pol^ in wild type R1158 and untreated and dox-treated (10 µg/ml) essential yeast strains was measured by Western blot analysis. Total proteins were extracted from equal numbers of yeast cells and analyzed by SDS/PAGE. Equal loading of total protein was verified by measuring Pgk1p levels.

**Figure 5:**
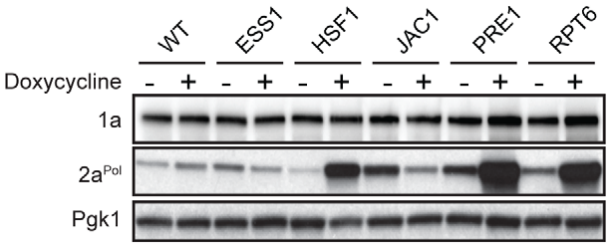
BMV 1a and 2a^Pol^ levels for dox-repressed essential yeast genes with inhibited BMV RNA replication. Accumulation of BMV 1a and 2a^Pol^ in wild type R1158 and untreated and dox-treated (10 µg/ml) essential yeast strains was measured by Western blot analysis. Total proteins were extracted from equal numbers of yeast cells and analyzed by SDS/PAGE. Equal loading of total protein was verified by measuring Pgk1p levels.

One possible reason for altered RNA replication is deregulation of viral protein accumulation. To test this, accumulation of BMV RNA replication proteins 1a and 2a^Pol^ were assayed by Western blotting. Addition of dox to growth medium had no detectable effect on BMV 1a and 2a^Pol^ accumulation in the wild type strain (Figs. 4 and 5). For 20 of 24 confirmed hits, BMV 1a levels in dox-treated strains were comparable to their untreated sample (Figs. 4 and 5). However, in P*_TET_*-*ADE13*, P*_TET_*-*DED1*, P*_TET_*-*PRE1* and P*_TET_*-*RPT6* dox-treated cells there was a detectable increase in 1a compared to the untreated strains (Figs. 4 and 5). Moreover, BMV 2a^Pol^ levels were significantly increased in P*_TET_*-*HSF1*, P*_TET_*-*PRE1* and P*_TET_*-*RPT6* dox-treated strains (Fig. 5). In P*_TET_*-*JAC1* dox-treated cells, 2a^Pol^ levels were elevated in the absence of dox, but reduced to near wild type levels in the presence of dox (Fig. 5). These results suggest that, with the potential exception of *HSF1*, *PRE1* and *RPT6*, viral protein regulation is unlikely to be the cause of altered viral RNA replication phenotypes upon depleting the products of the implicated genes.

## Discussion

We employed a high-throughput, systematic analysis of 741 dox-repressible essential yeast strains to identify 24 novel, essential host factors that alter BMV RNA replication. Our previous systematic analysis of ∼4,500 non-essential yeast deletion strains identified 99 genes that inhibited or enhanced BMV RNA replication [9]. Collectively, we have analyzed ∼93% of all yeast genes (∼5,800), and the 123 host genes identified to date that affect BMV RNA replication represent 2.3% of the yeast genome. As observed with tomato bushy stunt virus (TBSV) [38], another positive strand RNA virus, BMV replication is affected, directly or indirectly, by essential genes at a higher frequency (3.2%) than non-essential genes (2.2%).

For multiple reasons, our studies to date likely underestimate the number of host genes that contribute to BMV RNA replication. ∼60% of non-essential yeast genes are genetically redundant, meaning that the functions of many gene deletions are partially compensated for by other genes [39], [40]. Additionally, although the dox-repressible library is a powerful tool for analyzing the effects of essential genes on virus replication, over 70% of the strains in this collection exhibit growth defects in the presence of dox and 14% exhibit growth defects in the absence of dox [27]. Moreover, expressing viral components in some of these strains resulted in no or poor growth, interfering with meaningful analysis of these strains. Finally, in both the screens for non-essential genes [9] and essential genes (here), virus dependence on some host functions was likely masked by continuous expression of BMV replication proteins and RNA templates, compared to natural infections resulting from a single viral RNA template. This is analogous to studies in which high multiplicity of infection overcomes antiviral resistance in some cell lines [39], [41]. The lower confirmation rate for the 19 candidate genes that inhibited BMV-directed Rluc expression may be because these primary screen candidates included false positives arising from the tendency of essential gene depletion to produce non-specific inhibitory effects and greater variability in results, as is also noted above in the Results section. Additionally, translation and protein expression are the basis of our primary screen luciferase assays, whereas secondary confirmation by Northern blotting analyzes RNA levels normalized to 18S rRNA. Subtle differences (e.g., growth conditions) not controlled for by Fluc in the primary screen or 18S rRNA in Northern analysis may also contribute to the lower confirmation rate observed. Despite such limitations, this study identified 24 novel, essential host genes from various cellular pathways with potentially diverse roles in BMV RNA replication.

Enhanced BMV RNA replication upon repression of an essential gene suggests that, when present, the host factor contributes to an inhibitory response in a cellular process/pathway that competes with the virus. For example, of the 19 genes whose repression stimulated BMV RNA replication, 9 genes (*DHR2*, *ECM16*, *NOP7*, *PWP1*, *RIO2*, *RPA43*, *UTP4*, *UTP18*, *YGR251W*) are functionally associated with ribosome biosynthesis (Table 4). All of these genes perform or participate in ATP-dependent processes and depleting products of the implicated genes may result in an increased pool of energy and/or nucleotides available to the virus (Fig. 2, Tables 3 and 4). Alternatively, depleting these genes may alter the competition between viral and cellular translation. *UTP4* and *UTP18* function in rRNA processing, a cellular pathway that enhances TBSV replication (as shown by the effects of *UTP9* and *UTP15*) [38], but has the opposite effect on BMV RNA replication.

Depleting genes involved in processing cellular mRNA 3′ ends *(RNA15*), regulating transcription (*SPT6*), modulating cellular gene expression (*SWD2*), and controlling nucleocytoplasmic trafficking (*NUP57*) may increase the availability of ribosomes and/or alter the levels of specific proteins, preferentially stimulating BMV RNA replication (Fig. 2, Tables 3 and 4) by disrupting cellular pathways that would compete with BMV RNA translation or replication under wild type conditions. Thus, experimental depletion of these genes is in some ways analogous to the global shutoff of host mRNA pathways, employed by many mammalian viruses through diverse mechanisms [42], [43], [44]. Additional studies are necessary to define the role of these mRNA metabolism genes in BMV RNA replication.

In previous studies, we have observed that significantly disrupting the cell cycle and extended doubling times can non-specifically increase BMV RNA levels per cell, apparently because the virus has more time to accumulate RNA replication products prior to cell division (unpublished data). To avoid such false positives, we excluded from further analysis strains for which dox-treatment significantly slowed cell division (see Results above). Thus, although *SPC29* and *PFY1* encode proteins potentially related to cell division (respectively a spindle pole body protein and actin-binding protein) the doubling times of untreated and dox-treated P*_TET_*-*SPC29 and* P*_TET_*-*PFY1* cells were comparable and likely do not account for the increased levels of BMV RNA4 observed (Fig. 2. and Table 3). Alternatively, disrupting the cell cycle in P*_TET_*-*SPC29*- and/or P*_TET_*-*PFY1*-repressed cells may alter cell cycle signal transduction pathways or the localization of cellular factors that normally inhibit viral RNA replication.

Mutating general translation initiation factor *DED1* severely inhibits translation of BMV 2a^Pol^ from BMV genomic RNA2 in a fashion dependent on specific sequences in the RNA2 5′ noncoding region (NCR) [32]. Because the RNA2 5′ NCR was not present in the BMV expression constructs used in this study, we did not expect a detectable change in BMV RNA replication in the dox-repressed P*_TET_*-*DED1* strain. However, we observed an increase in RNA replication (Fig. 2 and Table 3), suggesting that dox-repression of P*_TET_*-*DED1* and global depletion of its gene products may have an alternative effect(s) on viral RNA replication compared to the previously analyzed *ded1-18* point mutant [32]. For example, under our screen conditions, repressing P*_TET_*-*DED1* may alter the production of viral or cellular proteins in ways that favor increased viral RNA accumulation. Further studies are necessary to define such potential additional role(s) for *DED1* in BMV RNA replication.

Reduced BMV RNA replication upon repression of an essential gene suggests that, when present, the host factor directly or indirectly facilitates viral RNA synthesis or accumulation. Interestingly, the five genes whose depletion inhibited BMV RNA replication (*ESS1*, *HSF1*, *JAC1*, *PRE1*, *RPT6*) have varied roles in protein stability or activation. For example, a small but reproducible (∼20%) inhibition of BMV RNA replication resulted upon dox-repression of P*_TET_*-*ESS1*, a peptidyl-prolyl cis-trans isomerase (PPI) (Fig. 3 and Table 5). Changes in isomerase activity can alter the structure, stability, or intracellular localization of client proteins [45], and deleting or mutating PPIs has variable effects on positive-strand RNA viruses [46], [47], [48], [49]. For example, knockdown of cyclophilin A and loss of its PPIase activity severely inhibits hepatitis C virus replication [46], [50], [51]. Conversely, siRNA-mediated knockdown of cyclophilin G mRNA stimulates hepatitis c virus replication [49]. Collectively, these findings suggest multi-faceted, complex roles for PPIs in positive-strand RNA virus replication.

*JAC1*, a member of the Hsp40/DnaJ family of proteins, encodes a specialized J-protein co-chaperone that assists Hsp70 in iron-sulfur (Fe-S) cluster biogenesis [52], [53]. Fe-S clusters are among the most versatile protein co-factors in the cell and participate in electron transfer, ribosome biogenesis, regulating gene expression and enzyme activity, and nucleotide metabolism [54], [55], [56]. The inhibition of BMV replication upon dox-repressing P*_TET_*-*JAC1* (Fig. 3 and Table 5) may result from negatively affecting the activation and/or function of one or more cellular or viral factors required for BMV RNA replication. Previously, we showed that *YDJ1*, another J-protein co-chaperone of Hsp70 and Hsp90, is required to activate the BMV RNA replication complex, likely through modulating BMV 2a^Pol^ folding or assembly into the complex [26]. Dox-repression of another heat shock protein, P*_TET_*-*HSF1*, inhibited BMV RNA replication by 84% (Fig. 3 and Table 5). Thus, our data suggest that BMV utilizes multiple members of the heat shock protein family to facilitate RNA replication.

Dox-repressing P*_TET_*-*PRE1*, a 20S proteasome core component, inhibited BMV RNA replication by 70% (Fig. 3 and Table 5). Similarly, repressing P*_TET_*-*RPT6*, one of six ATPases of the 19S regulatory particle of the 26S proteasome, resulted in an ∼90% reduction in BMV RNA replication (Fig. 3 and Table 5). Additionally, BMV 2a^Pol^ accumulation increased significantly in both P*_TET_*-*PRE1* and P*_TET_*-*RPT6* dox-repressed cells (Fig. 5). These results are consistent with previous findings that multiple non-essential ubiquitin-proteasome system components contribute to BMV RNA replication and that cells lacking *PRE9*, the only non-essential 20S proteasome component, also exhibit a substantial increase in BMV 2a^Pol^ levels [9]. Our prior data show that having a substantial excess of 2a^Pol^ shifts replication compartments from small spherular compartments to double membrane layers, but does not inhibit viral RNA replication [57]. Thus, the increase in 2a^Pol^ accumulation in dox-repressed P*_TET_*-*PRE1* and P*_TET_*-*RPT6* (Fig. 5) is not likely the cause of decreased RNA replication. However, prior results do not exclude the possibility that *PRE1* and *RPT6* depletion might affect BMV RNA replication by directly or indirectly modulating 2a^Pol^ localization, post-translational modification or interacting partners. The 26S proteasome localizes predominantly to the nuclear envelope-ER network [58], the site of BMV RNA replication [16], [59], and numerous viruses utilize the ubiquitin-proteasome system to facilitate infection or replication [60], [61], [62], [63], [64], [65]. Studies to define the specific role(s) of the ubiquitin-proteasome system in BMV RNA replication are ongoing.

With the exception of *NUP57*, *RNA15*, *SPC29* and *YGR251w*, each of the essential genes identified in this study have recognized orthologs in *Arabidopsis thaliana* (http://www.arabidopsis.org/ and http://ppod.princeton.edu/), presenting the possibility that similar genes could function in BMV RNA replication in its natural plant hosts. For example, we have shown here that dox-repression of Hsp70 cofactor *JAC1* or heat shock protein transcription factor *HSF1* significantly inhibits BMV RNA replication. *Arabidopsis* encodes 18 Hsp70 family members and Hsp70s have been shown to affect the replication of other (+)RNA viruses in plants, including TBSV and turnip mosaic virus [66], [67].

Similarly, *PRE1* (*PBD1* and *PBD2* in *Arabidopsis*) and *RPT6* (*RPT6a* and *RPT6b* in *Arabidopsis*) are essential components of the highly conserved 26S proteasome and recent results from our laboratory show that BMV RNA replication in yeast and plant cells depends critically on the ubiquitin-proteasome pathway (B. Gancarz and P. Ahlquist, unpublished results) [9], [68].

In summary, our high-throughput analysis of essential yeast genes identified a diverse set of host factors that affect BMV RNA replication and significantly expanded our knowledge of cellular pathways utilized by BMV. Additional studies both in yeast and in BMV's natural plant hosts should reveal how these host factors affect the virus and provide new insights to host cell function and virus-host interactions. Although targeting some essential genes may result in deleterious effects on cells or patients, focusing on the relevant cellular pathways, rather than only individual genes, may overcome such issues. For example, two of the essential genes with the most substantial inhibitory effect on BMV RNA replication, *PRE1* and *RPT6*, are proteasome components, while other proteasome components are not essential (e.g. *PRE9*, identified in previous screen). Proteasome inhibitors have antiviral activity against multiple diverse viruses (including herpes simplex virus, hepatitis B virus, and HIV, among others), have been through multiple clinical trials, and are already approved for use in patients for some purposes. Many of the genes identified function in pathways utilized by other viruses and thus may present potential cellular targets for developing broad-spectrum antivirals.

## Supporting Information

Table S1 **Dox-repressible essential yeast strains that did not transform with BMV expression plasmids after repeated attempts.**
(XLSX)

Table S2 **Number of dox-repressible essential yeast strains excluded at each pass and time point due to growth or Fluc values.**
(XLSX)

Table S3 **Dox-repressible essential yeast strains excluded from reporter gene-based primary screen analysis due to growth and/or Fluc value.**
(XLSX)

Table S4 **Reporter gene-based primary screen data for dox-repressible essential yeast genes included in final data analysis.**
(XLSX)

Table S5 **Negative-strand RNA3 levels for essential yeast genes confirmed to enhance BMV RNA accumulation in secondary validation testing.**
(XLSX)

Table S6 **Negative-strand RNA3 levels for essential yeast genes confirmed to inhibit BMV RNA accumulation in secondary validation testing.**
(XLSX)
